# Health professionals perception and beliefs about drug- related problems on polymedicated older adults- a focus group study

**DOI:** 10.1186/s12877-020-01972-3

**Published:** 2021-01-07

**Authors:** Ana Isabel Plácido, Maria Teresa Herdeiro, João Lindo Simões, Odete Amaral, Adolfo Figueiras, Fátima Roque

**Affiliations:** 1 Research Unit for Inland Development, Polytechnic of Guarda (UDI-IPG), Avenida Dr. Francisco Sá Carneiro, n. ° 50, 6300-559 Guarda, Portugal; 2 Institute of Biomedicine (iBiMED) and Department of Medical Sciences, University of Aveiro, Campus Universitário de Santiago, 3810-193 Aveiro, Portugal; 3 Institute of Biomedicine (iBiMED) and School of Health Sciences, University of Aveiro, Campus Universitário de Santiago, 3810-193 Aveiro, Portugal; 4 Health Sciences School, Polytechnic of Viseu, R. D. João Crisostomo Gomes de Almeida, n.° 102, 3500-843 Viseu, Portugal; 5 Department of Preventive Medicine and Public Health, Faculty of Medicine, University of Santiago de Compostela, Santiago de Compostela, Spain; 6 Consortium for Biomedical Research in Epidemiology and Public Health (CIBER en Epidemiología y Salud Pública-CIBERESP), Santiago de Compostela, Spain; 7 Institute of Health Research of Santiago de Compostela (IDIS), Santiago de Compostela, Spain; 8 Health Sciences Research Centre, University of Beira Interior (CICS-UBI), Av. Infante D. Henrique, 6200-506 Covilhã, Portugal; 9 Escola Superior de Saúde, Instituto Politécnico da Guarda Rua da Cadeia, 6300-035 Guarda, Portugal

**Keywords:** Health professionals, Polypharmacy, Drug-related problems, Medication management, Older adults

## Abstract

**Background:**

Polymedicated older patients are at greater risk of suffering from adverse events. For this reason, the detection of both inappropriate polypharmacy and polypharmacy-associated Drug-Related Problems (DRPs) are essential to improve the health and wellbeing of older adults and to reduce healthcare costs. This work aims to explore health professionals’ perceptions and opinions about polypharmacy and the handling of medicines by polymedicated older adults.

**Methods:**

Thirteen focus groups with 94 health professionals (20 community pharmacists, 40 general practitioners and, 34 nurses) were conducted in primary healthcare centers of the center region of Portugal. Participants were asked to discuss their perceptions and beliefs concerning DRPs in polymedicated older adults. The sessions were audiotaped. After the transcription and coding of focus group sessions, a thematic analysis was done.

**Results:**

The following four main themes emerged from the 13 focus group sessions: poor compliance and polypharmacy- A perpetuated vicious cycle, organization of the healthcare system, interaction and communication between the health professionals, and strategies to prevent inappropriate polypharmacy.

**Conclusions:**

The lack of both an efficient network of information and Interaction and communication between Health professionals makes the detection and/ or prevention of polypharmacy in older adults difficult. The implementation of new models to manage and/or prevent polypharmacy based on health professional perception and beliefs is essential to prevent DRPs and improve compliance among older adults.

**Supplementary Information:**

The online version contains supplementary material available at 10.1186/s12877-020-01972-3.

## Background

The world population is aging, and it is expectable that by 2050 one in six people will be over the age of 65 [1]. Age-related loss of resilience and the progressive decline across physiologic systems predispose older adults to multiple chronic diseases that often require the prescription of multiple medicines [2, 3]. Despite the efforts of health professionals (HPs) to balance prescription and the multiple comorbidities, drug-related hospital admission of older adults still ranges from fifteen to 30 % of all hospital admissions [4].

United Nations defined as a worldwide priority “*ensure healthy lives and promote well-being for all at all ages*” [1, 5].

According to a recent study, although being one of the most aged countries in the world, only 9% of the Portuguese older adults were considered healthy and, almost 50% of them had difficulty complying with their medication regime [6, 7].

In the last decade, the studies on Drug-Related Problems (DRPs) have focused on medication errors committed by HPs and attributed medication errors to mistakes in prescribing, preparation, or dispensing [8]. To our knowledge, this is the first focus group (FG) study that explores, in the same FG session, the beliefs and concerns of pharmacists, general practitioners, and nurses in primary healthcare centers, regarding polypharmacy in older adult patients, as well as their opinions on how to approach or prevent DRPs.

FG studies can be a valuable tool because they allow the identification of all dimensions of a problem, even those that are unexpected [9, 10]. Accordingly, this study aimed to explore the health professionals’ perceptions and opinions regarding both polypharmacy and the managing of medicines by polymedicated older adults of the center region of Portugal that is the second region with the highest aging index (201.4) in the country [11].

This study provides valuable data for the implementation of a new healthcare model of polypharmacy management in primary healthcare systems.

## Methods

An exploratory qualitative study using an FG approach was designed to explore the perception of primary care HP, community pharmacist (CP), general practitioner (GP), and nurses about polypharmacy in older patients. It was asked the collaboration of healthcare center directors to participate in the study collaborating in the invitation of HPs. FG sessions were moderated by a PharmD/ Ph.D. researcher (FR), following a guide based on a systematic review study and drawn up by a group of experts in pharmacology and epidemiology (Additional file 1: Appendix 1) [8]. The moderator interfered only if a topic was not directed or if the discussion came to a stoppage. The Consolidated criteria for Reporting Qualitative research (COREQ) were followed (Additional file 1: Appendix 2) [12].

### Setting

The FG sessions took place at 13 public health centers coming under the Centre Regional Health Administration (Administração Regional de Saúde do Centro) and encompassing a total of 40,835 registered older patients (age ≥ 65 years).

### Holding of focus group sessions

For each FG session were invited at least 2 CP, 2 nurses, and 2 GP. FG sessions were conducted from May to October 2018 and lasted for 60–90 min. They were carried out until saturation of information was reached on the research questions. Before the beginning of each session, the moderator reminded the participants of the study goals and the fact that audio records were being made of the sessions. The moderator ensured the participants that the content matter would remain confidential at all times and that the data would be processed without the identification of participants. All participants signed an informed consent form before participation. The participation of the HPs was voluntary, without any incentives.

### Analysis

All the sessions were transcribed and coded by a researcher (AIP). Each session was codified with the acronym FG followed by an alphanumeric character. The participants of each session were also codified with the acronym of their FG followed by the acronym CP for community pharmacists, GP for general practitioner, or N for nurses and an alphanumeric character (e.g. FG1CP1). To guarantee trustworthiness, one month after the last hearing, the tape was listened, once again, and the transcription content was revised. A thematic analysis was done, following the methodology described by [13]. Braun and Clarke 2006) define thematic analysis as “*a method for identifying, analyzing and reporting patterns (themes) within data”. “It minimally organizes and describes data set in (rich) detail”* [13]*.* In a first approach, and to allow a better understanding of the content of FG all the transcripts were read multiple times by two researchers. After that, initial codes were generated and grouped into themes. Then themes were revised, and data interpreted and discussed by the team, before emerging the overarching concepts. NVivo qualitative data analysis software (QSR International Pty Ltd. Version 12, 2019) was used to help with the organization and analysis of the data.

## Results

Overall, 13 FG were conducted with a total of 94 HPs enrolled (40 GP 20 CP and 34 nurses). The sociodemographic characteristics of the participants are summarized in Table 1.

**Table 1:** Participants characteristics

General Practitioners		
Female (*n* = 16)	Male (*n* = 24)	Total (*n* = 40)
Mean age (min; max)		
46.6 (27–65)	53.0 (28–66)	50.5 (27–66)
Mean years of experience (min; max)		
22.2 (1–31)	25.3 (2–40)	23.7 (1–40)
Nurse		
Female (*n* = 32)	Male (n = 2)	Total (*n* = 34)
Mean age (min; max)		
45.1 (32–63)	42.0 (38–46)	44.9 (32–63)
Mean years of experience (min; max)		
25.7 (3–41)	11.5 (4–19)	18.6 (3–41)
Community Pharmacist		
Female (*n* = 17)	Male (n = 3)	Total (*n* = 20)
Mean age (min; max)		
40.8^a^ (23–58)	30.7 (24–36)	39.1^1^ (23–58)
Mean years of experience (min; max)		
15.4 (1–34)	7.0 (1–12)	11.2^1^ (1–34)

^a^Two missing values

Qualitative analysis resulted in four major themes: “poor compliance and polypharmacy- A perpetuated vicious cycle”, organization of the healthcare system, interaction and communication between health professionals, and strategies to prevent inappropriate polypharmacy (Table 2).

**Table 2:** Major themes from focus groups

Theme		Subthemes	Coding concepts
“Poor compliance and polypharmacy- A perpetuated vicious cycle”.		Polypharmacy	Perception
		Socioeconomic factors	Familial context
			Economic factors
		Knowledge	Literacy
			Identification of medicines
			Duration of treatment
		Compliance	Adverse effects
			Priority
			Patients beliefs
		Deprescribing	Difficulties
		Patients-HPs communication	Lack of communication
		Influencers	TV supplements
			Neighbours medication
		Herbal products	Interactions
		Medicines managing	Handling
			Generic medicines
Organization of healthcare system	Medication management directives	Healthcare directives	Prescribing guidelines
			Patients empowerment
	Clinical appointments	Clinical appointments	Lack of time
Interaction and communication between Health professionals	External responsibility	General practitioner’s -Community pharmacists	Trust/mistrust
		General practitioner’s -Specialist physicians	
	lack of time to communicate	General practitioner’s -Specialist physicians	Lack of communication
		General practitioner’s -Community pharmacists	
		General practitioner’s -Specialist physicians	Multiple prescribers
Strategies to prevent inappropriate polypharmacy		Prescribing	managing
			Generic medicines
		Promotion of compliance	Empowerment of patients
			Support teams

### Poor compliance and polypharmacy- a perpetuated vicious cycle

According to HPs, aged-related comorbidities prone older adults to multiple prescriptions, and, for this reason, polypharmacy is an unavoidable consequence of aging.

“*The presence of multiple comorbidities is a normal consequence of ageing and for this reason polypharmacy in older patients is common practice*”, FG5GP1.

HPs also perceive that polypharmacy and poor compliance are two faces of the same coin, because polymedicated older adults have difficulties to comply with the therapeutic regime, hampering the achievement of clinical outcomes and leading GPs to prescribe one more medicine.

*“Polypharmacy leads to poor compliance and the poor compliance conduce to polypharmacy …*. *Because if they do not adhere to a therapeutic regime … we prescribe for one situation, after that for another proble*m”, FG13GP1.

Because older adults value medicines and admit that they are essential to promote wellbeing, HPs believe that most of the time, medication errors are unwitting and committed due to the lack of knowledge of older adults.

*“Polypharmacy is closely related to literacy”,* FG11GP1.

From this point of view, lack of literacy, sociocultural factors, such as the absence of family and/or relatives to take care and help with the management of medicines are the main contributors to medication errors.

*“The problem of polypharmacy is the loneliness of the older adults that do not have any young familiar near to help*” FG2CP2.

During their daily routine, HPs perceived that because older patients recognize medicines by the color and/or shape of the pill, duplication mistakes, mainly due to generic medicines confusion, or taking medicines, which belong to others, can easily happen.

*“One older woman brought to the healthcare center, both their medicines and the medicines of their husbands because they do not recognize their pills”,* FG6N3.

*“There are several generic medicines for the same active substance (from different holders) and older patients identify the medicines by the color of the tablet … /if a doctor prescribes to him another medicine or deprescribes a medicine if they have the pill at home, they will continue to take it”,* FG1CP1.

During FG sessions, it was also perceived that older adults have some difficulties to understand the duration of treatments, and for this reason, they tend to prolong the treatments for more time than is recommended by the GP. These mistakes are frequently detected in medicines prescribed for an acute episode that requires an emergency room visit.

*“After an emergence visit, we frequently observed that older patients, come to the pharmacy with a specific medicine, and at a certain point they do not know if they should stop or not take that medicine, and for this reason, they continue taking it”* FG1CP1.

According to HPs, difficulties related to the use of some medicines, such as inhalators, can influence the efficacy of treatments.

*“There is a lot of confusion, for example, inhalators, they have difficulties, the device has a counter but they don’t know, and sometimes they arrive at the pharmacy and told us that the inhaler is empty but is new, they do not use it*”, FG2CP1.

According to HPs, the desire of older adults to achieve wellbeing makes them easily influenced by the neighbors or even by television commercials, potentiating the consumption of over-the-counter (OTC) drugs, supplements, or even herbal products. These substances besides interfering with chronic medication might be contraindicated for their health problems.

*They are easily influenced by their neighbors, which say: I took this pill and I am feeling very well” FG3GP1.*

*“Hide a lot of information, sometimes there is no medical prescription, it was a recommendation of the neighbors” FG1CP1.*

*“Several patients buy on TV calcium pills” FG1FGP1.*

HPs believe that polypharmacy per se is a contributor to poor compliance because older adults take so many medicines that they easily withdraw some that they believe to be less important.

“*Sometimes some patients complain that they take more medication than food*”, FG1CP1.

According to HPs perception, the huge number of pills throughout the day prompts older adults to skip a dose, to give up some medicines that they undervalue, or that they believe might cause adverse effects.

*“Polymedicated patients always try to remove one or another pill that they believe does not affect”,* FG3GP1.

*“If someone refers to the side effects of statin, patients automatically stop taking”,* FG8GP1.

During their practice, GPs perceived that fake beliefs of older adults make the deprescription of medicines difficult.

*“Deprescribing some medicines it was almost impossible, for example, trimetazidine is very, very hard”,* FG3GP1.

“*If I want to deprescribe pills that the patient is taking to 30 or 25 years, with which they felt good … we understand that it’s causing more harm than good … it is very difficult”,* FG5GP2.

Another important compliance-influence factor that emerged during the FG discussions was the price of medicines.

“The *price of the medicines also contributes to poor adherence*” FG1GP1.

According to HPs, patients with economic problems, adjust the therapeutic regimen to spend less money.

“*… a large number of older adults that take oral anticoagulants, the new ones that are more expensive, sometimes instead of taking two pills only take one”* FG4GP3.

However, some HPs also pointed out that the Portuguese National Health System (NHS) is predominantly financed by taxes and some out-of-pocket payments that include co-payment for a wide range of services, though there are income-based exemptions population groups (and certain medical conditions). Portuguese older adults with an average salary of 1.5 times below the value of the social support index are exempt from co-payment for any publicly provided services. For these reasons, some HPs affirms that sometimes the low price of medicines promotes the wastage of health resources.

“Low *price of some medicines, namely insulin promotes the wastage of health resources because patients tend to open new insulin before the first finished, and put the older on the garbage*” FG2CP1.

During FG sessions, it was perceived that the lack of communication between patients and physicians is not only an important polypharmacy-related factor but also an influencer of compliance. Because patients do not report to their GP either the specialist appointments that they had or the new prescriptions or other products that they take, inappropriate polypharmacy is harder to detect.

“… *older adults do not report their specialist/ emergency room visits to the GP, for these reasons’ GP has difficulties in detecting this inappropriate polypharmacy*” FG1GP5.

*“Teas herbal products and other blend beverages that patients buy here or there because of their health problems... One for the gallbladder, one another to the head, other to the kidney, and all of these substances have an active substance. All of them can cause drug interactions …. That we cannot control*” FG5GP1.

### Organization of the healthcare system

HPs perceived that the organization of the healthcare centers doesn’t simplify the identification of DRPs. Within this theme, two subthemes emerged, i.e. “Medication management directives” and Clinical appointments.

#### Medication management directives

HPs believe that the lack of centralization of patients chronic medication compromises clinical outcomes and promotes duplication mistakes.

*“The lack of centralization of chronic medication management is a problem, because we have many vulnerable older patients … , who have several physicians’ appointments, consequently to many prescribers, both in the public and private sector. These physicians have very great freedom to prescribe, which makes that the physician where all the information should converge, theoretical the GP, have difficulties in handling and evaluate all the prescribed medication …. And this then generates situations such as polypharmacy, adverse reaction, drug interaction”,* FG1GP1.

NHS guidelines recommend that HPs “*must privilege the use of electronic means to support the processes of prescribing, dispensing and billing of all types of medicines, as well as health products*” [14]*.* For this reason, the prescription and the treatment guide are sent by SMS (short message service) to the mobile phone*.* According to HPs, these directives make the empowerment of patients on medication management difficult*.*

*“I think, there is some pressure for physicians to stop printing the treatment guide …. The older population needs the guide treatment written in a paper”,* FG5N1*.*

*“Into the older population that has a cell phone, some do not know how to use it, and when they want to see the treatment guide sometimes, they press the wrong button and once upon a time a treatment guide, they delete all”,* FG5N2.

#### Clinical appointments

According to HPs, the short time of the clinic appointments’ hampers not only the therapeutic review process but also the review of the handling of medicines by older adults.

“*The 15 minutes of clinic appointment turns out to be little to explore these issues”,* FG13GP2.

HPs also refer that the lack of time during clinic appointments is an important polypharmacy-related factor that sometimes is undervalued*.*

*“It takes time to see all … and we do not have time” FG2GP5.*

### Interaction and communication between health professionals

Within this theme, two subthemes emerged, i.e. lack of time to communicate and external responsibilities.

#### Lack of time to communicate

HPs believe that in some healthcare centers the interaction among HPs promotes the detection of DRPs.

*“… yesterday, a pharmacist called to tell me that a patient bought a statin different from what I had prescribed, I appreciate that”*, FG4GP3.

However, when managing patients’ therapeutics, this is not always a reality.

“*There are units that make protocols with local pharmacies … the problem is the lack of time*” FG1GP1.

*“… must-have big management of medicines, and for this is necessary do therapeutic revisions and presently GP, perhaps because they lack time, they are not doing it”, FG2CP3.*

In Portugal, primary healthcare centers are the gatekeeper of the NHS, so, whenever a patient needs a specialist appointment, the GP requests the appointment and sends all the clinical processes of the patients to the specialist. During FG sessions, it was perceived that it is hard to obtain the return information, suggesting that there is a lack of communication between GPs and the other physicians.

“… *we are obliged, and even if we were not, we always send complete information with the medication with everything, and then we never get the return*”, FG4GP1.

“*Sometimes happens patients are taking an active substance for hypertension prescribed by the GP, therefore prescribed by myself, in the meanwhile, for any reason they go to the emergency service and comes to the home with other hypertensive medicines from another chemical group, which must not be taken with the previously prescribed hypertensive. Because physician, that works at the emergency service, did not take the trouble to see the chronic medication of the patients, the patients take the pills*”, FG10GP1.

This lack of time to communicate becomes more demanding in circumstances such as the deprescribing process.

*“I do not feel comfortable to remove some medicines, a cardiologist appointment, patients expect eternity, so they go to a private clinic and, if I call the cardiologist, he will say if you want to remove the medicine do it, but is your responsibility”,* FG10GP5.

#### External responsibilities

The lack of time to communicate promote misunderstandings among HPs and they tend to assign responsibility to others. According to GPs, CPs are not doing their job because before selling a medicine they should ensure that patients know how to use the medicines.

*If patients have, doubts are pharmacist faults …. They must explain well because they sell the medicines, they have all the material”,* FG10GP3.

GPs also affirm that some duplication mistakes occur, because CPs replace the original medicines, which they prescribed, with generic medicines. On the other hand, CPs feel undervalued by physicians who tend to forget their role and the fact that their proximity to older patients makes them know patients’ needs better. Moreover, CPs affirm that when selling a generic medicine, they do it responsibly and because they know the economic context of the patient.

*“What happens is that GP does not have the perspective of the price of medicines, and they prescribe medicine and, when the patient came to the pharmacy they ask us if we do not have a cheaper medicine, “I do not have money to buy this …*” */ we are not changing the therapeutic, we are first helping the patients”* FG10CP2.

### Strategies to prevent inappropriate polypharmacy

According to HPs, polypharmacy could be reduced if GPs were able to act as the manager of all prescriptions, i.e. GP should have the opportunity to validate/ or not a prescription, prescribed by other physicians, before the dispensing.

*“All the prescription must have to be authorized and validated by the GP, that managing and planning the health of the patient”, FG1GP1.*

During FG sessions it was perceived that HPs admitted that the introduction of the.

Electronic prescription of medicines (PEM) promotes the managing of medicines; however, this platform had some gaps that make the detection of DRPs a hard task. GPs affirmed that when using PEM, they find difficulties in updating chronic medication. They also believed that all HPs should have access to this platform. HPs believe that the limited access of PEM to the nurses hampers their role in DRPs detection. Moreover, CPs could also have a more active role in DRPs detection, if they could access PEM. CPs could relieve the burden of primary healthcare centers in terms of time and duration of clinical appointments, through the opportunity to renew the chronic medication.

*“If the PEM allows the update of chronic medication and if pharmacists could access the PEM, the pharmacist can make the renewal of the chronic medication and, this in turn, relieve the burden that physicians have in terms of patient appointment” FG1GP1.*

To avoid DRPs related to duplication of medicines, HPs suggested that the pharmaceutical industry should agree to standardize the boxes of medicines and even, if possible, the color and form of pills by active substance.

*“The boxes of the same active principle should have the same color …”* FG1GP2.

“the pills should also be standardized in terms of shape and color, FG1N1.

Lastly, health professionals believe that to decrease DRPs it is essential to support, empower the patients and promote health literacy.

*“The ideal would be to have a support team not only to make the dressings and emergencies but also to visit the needy patients that live alone. Older adults often do things on their way because they don’t want to ask for help and they don’t have support either... The support would be to try to understand if the medication is being well managed”,* FG5N1.

*“The user comes to the health center, takes the prescription I can even know if he raised the boxes in the pharmacy, but on the home visit, I can find a warehouse of boxes of medicines”, FG5GP1.*

*“Promote health literacy”,* FG3GP2.

*“The awareness campaigns could be a good help to patients and healthcare professionals*”, FG5N1.

## Discussion

Worldwide, one of the consequences of the demographic trends in ageing was the increasing number of ageing-related comorbidities. Because of that, the implementation of new models to deal with the consequences of polypharmacy is fundamental not only to ensure the welfare of the older population but also to guarantee the efficacy of healthcare systems. From this point of view, understanding HPs perceptions and beliefs is fundamental.

To our knowledge, this is the first FG study that encompasses a multidisciplinary team of HPs (physicians, nurses, and pharmacists in the same FG session), who daily take care of polymedicated older adults, to explore their perception and opinions regarding polypharmacy-associated DRPs in older adults.

HPs believe that the economic/ social context of older adults influences their behavior, and, in cases of low literacy, low mensal incomes, and lowliness, it can trigger the occurrence of DRPs. The short time of clinical appointments, the lack of time to communicate and network between all HPs, the poor communication between HPs and patients were identified as the main gaps in the healthcare system that difficult for both the managing of polypharmacy and the detection of DRPs. The decrease of the gaps mentioned above and the empowerment of polymedicated older adults can have positive effects on patient health outcomes through the improvement of compliance and the reduction of DRPs.

During FGs sessions, we perceived that overall; the different HPs have complementary and frequently similar answers. However, HPs tend to assign responsibilities to other HPs, CPs affirm that GPs are not doing their work on the therapeutic review, and GPs believe that CPs are not due to their work on counselling patients. CPs also affirm that they do their best the managing their patients, and they make an effort to manage each patient according to patients’ socio-economic reality and argue that GPs do not value their work.

Due to the overload of the Portuguese NHS, patients wait months or even years for a specialist appointment. This waiting time together with the overwhelmed that Portuguese physicians’ feelings are, according to our perception, the main reasons for the inappropriate communication between physicians and a source of misunderstanding. This situation becomes more demanding because, in some situations, patients instead of waiting for a specialist appointment opt to go to a private clinic and, they do not report these appointments to GP.

Our study participants consider that the presence of multiple comorbidities associated with the fact that the guidelines are based on single chronic diseases makes polypharmacy a recurrent issue among older adults. Moen et al. [15] reported that although physicians trust in guidelines, sometimes they felt insecure because older people are underrepresented in the studies on which the guidelines are based. For this reason, physicians perceive that following guidelines for some diseases, such as cardiovascular diseases, might prompt the occurrence of polypharmacy [8]. Our results also showed that polymedicated older adults more easily fall into a “prescription cascade”. This phenomenon was observed in clinical practice for the first time by Rochon et al. in 1995 [16, 17]. During our study, HPs also reported that polypharmacy potentates the occurrence of interactions and they felt that they are “walking on thin ice”. Our study demonstrated that media, websites and even patients’ peers frequently gave patients “a false awareness of knowledge about medicines and because of that, patients tend to pressure HPs to obtain the products that they wish, and not what they need [18, 19]. A recent FG study [20] observed that older patients trust in HPs, and believed that they play a determinant role to ensure the correct managing of medicines and decreasing DRPs. However, older adults also admitted that sometimes they decide not to comply with the therapeutic regime, due to influences of family and friends, television commercials, fear of an adverse reaction, etc., and they do not share their decision with health professionals [20]. These observations are following our study since HPs perceived that older adults hide a considerable amount of information regarding the adaptation of the therapeutic regimen according to friend opinion and the consumption of herbal products. Both HPs and patients [20] perceived that: (i) the influence of friends and television advertisements potentiate the consumption of supplements and other products, such as herbal products, which might prone them to quit their prescription medicines; (ii) the lack of knowledge of older patients and the absence of caregivers to help with the management of medicines improve both medication errors and poor compliance with consequently poor health outcomes; (iii) Because older patients recognize their medicines by color and shape, the lack of standardization of dosage forms, color and packaging, the change of trade mark drugs for generic drugs or changes of generic drugs holders, is enough for older adults to commit treatment duplication errors when they handle generic medicines. This observation was found in a previous study [21], (iv) older patients attach great value to their medicines, however, when they take many medicines, they weigh the risks and benefits and might stop taking some, (v) older patients trust in their HP, but they believe that there is no need to share the use of other products (like OTC) with the GPs; this purposeful omission of information influences treatment efficacy and makes the detection of DRPs difficult. According to Pound et al. [22] patients believe that flexibility in taking their medication allows life to continue without too many disruptions.

Safran et al. [23] reported that older adults with fewer resources are less compliant and tend to skip doses to make their medicines last longer. Einav et al. [24] observed that individuals tend to consume less healthcare when they are required to pay more for it out of pocket; suggesting that moral hazard in health insurance exists. Moreover, an Irish study observed that insured older adults tend to experience more polypharmacy than not insured older adults [25]. In older adults from Korea, it was observed that the low-income status associated with the coinsurance payment policy could trigger a large medication cost burden [26]. To avoid a medication cost burden, the co-payments on Portuguese NHS include exceptions and people with certain medical conditions and older adults with low incomes are exempt from co-payment for publicly provided services [27–29]. Moreover, the co-payments for medicines ranging from 15 to 90% of co-insurance are applied according to their therapeutic value. In some of the most aging cities of Portugal, regional governments implemented measures to support the cost of medicines for older adults with low incomes [27–29]. For these reasons, we believed that low incomes could influence therapeutic adherence, but the lack of health literacy and the poor empowerment of the patients influence even more. Previously studies reported that there are problems when transferring information between healthcare facilities and that this problem is critical when they intend to deprescribe medicines [30–33]. For this reason, the development of deprescribing approaches is essential for optimizing medication management and reducing polypharmacy in older adults [34, 35].

We believe that the development of health policies directed to polypharmacy as well as the implementation of computerized clinical decision support systems [36] will not only facilitate the managing of polymedicated older adults by GP but also avoid DRPs such as medicines duplication and drug interactions. As previously reported [37] we believe that CPs could also have a more active role not only in minimization of duplication mistakes due to patients’ confusion, but also to promote the trust of the patient in generic medicines and perhaps improve medication due to the lower price of generics.

This study has some limitations linked to the chosen methodology. FG participants were invited/ selected by healthcare center directors, the results cannot be generalized to the whole population, timid participants have more difficulty to share their perceptions, and participants may follow the general trend to give similar answers. This methodology’s generalizability is best judged in both exploring the perception of the participants and to favor the arising of a new topic [38]. This methodology also has the advantage to promote the interaction of all participants, facilitating the communication of the timidity participants. Because the convenience sampling used, selected based on the individual characteristics of the overall population, this methodology can contribute to helping researchers gain a greater understanding of the research question rather than from a statistically representative of a broader population [39]. To decrease limitations associated with FG methodology, FG sessions were undertaken at several healthcare centers located across inland and coastal areas of the Portugal center region [40]. All sessions were moderated by the same moderator.

## Conclusions

Portuguese GPs feel overwhelmed and for this reason, they do not have time to revise the therapeutic of their patients. To minimize DRPs-associated polypharmacy in older adults, GPs, nurses, and CPs should interact to facilitate the therapeutic review. The strengthening of communication among HPs, between HPs and patients and the improvement of literacy of older adults regarding the managing of their medicines is essential to enhance health outcomes among polymedicated older adults.

In 2018, our group initiated the MEdElderly project to improve drug managing among the older population of Portugal’s center region. The results of this FG study were useful to design an educational intervention that aims to address the main DRPs observed in older adults, demystify false beliefs and promote the correct management of medicines.

## Supplementary Information

**Additional file 1.**

## Data Availability

The authors confirm that the data supporting the findings of this study are available within the article.

## Abbreviations

CP: Community pharmacist
DRPs: Drug-Related Problems
FG: Focus Group
GP: General Practitioner
HPs: Health Professionals
NHS: National Health Service
OTC: Over-the-counter
PEM: Electronic prescription of medicines
